# The digitisation workflow of the herbarium of the State Museum of Natural History of the NAS of Ukraine (LWS)

**DOI:** 10.3897/BDJ.13.e148861

**Published:** 2025-03-28

**Authors:** Andriy Novikov, Viktor Nachychko

**Affiliations:** 1 State Museum of Natural History of the NAS of Ukraine, Lviv, Ukraine State Museum of Natural History of the NAS of Ukraine Lviv Ukraine; 2 Ivan Franko National University of Lviv, Lviv, Ukraine Ivan Franko National University of Lviv Lviv Ukraine

**Keywords:** herbarium, natural history collections, digitisation workflow, imaging, data mobilisation

## Abstract

The digitisation workflow currently applied at the Herbarium of the State Museum of Natural History of the National Academy of Sciences of Ukraine (LWS) differs from other similar by cascade ('object-to-data-to-image') multilevel organisation. Its application is predicted by the need to preselect specimens by taxon and region, as well as by batched digitisation, which occurs with significant interruptions. Focusing on certain taxonomic groups from specific regions allows us to digitise specimens that could be more valuable for early scientific processing. At the same time, the herbarium benefits from such a digitisation model by revising the existing collection classification and keeping the initial ID system. The presented digitisation workflow can be easily reproduced in any herbarium with a limited budget. The purpose of this paper is to provide detailed description and schemas of the principal digitisation stages applied at the LWS Herbarium and to briefly discuss the steps crucial for a successful result. Provided information should help to maintain the digitisation and choose appropriate equipment and materials. We can conclude that, despite its general complexity, the described workflow demonstrated itself as viable and relevant due to its robust design and focus on data quality. Despite a focus on specialists' involvement, it maintains flexibility that allows combining volunteers and, if needed, outsourced efforts. Moreover, its modularity promotes independence of principal digitisation stages and allows long interruptions between the digitisation batches.

## Introduction

Herbarium digitisation is a crucial modern task providing numerous benefits such as biodiversity data mobilisation and remote access to collections (Cantrill 2018, Borsch et al. 2020, Nieva de la Hidalga et al. 2020, Powell et al. 2021, Davis 2023). Despite the increased popularity of citizen science and specialised biodiversity platforms (e.g. iNaturalist) allowing the direct registration of plant occurrences in nature, collecting the herbarium specimens remains essential. Herbarium specimens serve as a source of verifiable and falsifiable scientific evidence, which can be limited for published citizen-science observations, even if they are supported by photographic content (Soltis 2017, Funk 2018, Heberling et al. 2019, Heberling 2022, Park et al. 2022, Niedzielski and Markiewicz 2023, Eckert et al. 2024). For example, the micromorphological or anatomical data cannot be received and DNA cannot be extracted without a physical specimen. It has also been shown that herbarium data better (compared to the citizen-science data) reflect the taxonomic, phylogenetic and functional diversity of individual regions and allow for much better species distribution modelling, with fewer taxonomic and geospatial errors (Eckert et al. 2024). With the advent of remote access to herbarium collections and the development of digital image processing technologies, new approaches to digital data analysis are emerging, including automatic or semi-automatic species identification (Carranza-Rojas et al. 2017, Shirai et al. 2022, Takano et al. 2024), morphological and phenological analyses (Younis et al. 2018, Goëau et al. 2020, Hodač et al. 2024) and georeferencing and geotagging (Beaman and Conn 2003, Magdalena et al. 2018, Nowak et al. 2021). Despite impressive progress in the digitisation of the world-leading collections and rapid development and implementation of AI technologies in processing the derivate herbarium data (Pearson et al. 2020, Goëau et al. 2021, Shirai et al. 2022), the herbaria with limited financial and staff facilities are still in search of cost-efficient and simple digitisation solutions (Allan et al. 2019, Takano et al. 2019, Santos et al. 2020, Borsch et al. 2020)

The Herbarium of the State Museum of Natural History of the NAS of Ukraine (LWS) has been established in Lviv City since 1832. It is the third oldest and seventh richest herbarium in Ukraine (Shiyan 2011). The LWS Herbarium includes ca. 147,000 specimens, most of which are represented by specimens of vascular plants. Digitisation at the LWS Herbarium was launched in 2012 and occurred sporadically, with interruptions caused mainly by a lack of financial support. Hence, at different times, digitisation focused on different taxonomic groups and aimed to solve various tasks. Such variation determined the kind of applied workflow and final product (i.e. mobilised data and/or digital images) dissemination. In particular, initially, the type specimen digitisation was outsourced to the Herbarium of the Ivan Franko National University of Liviv (LW) (Tasenkevich et al. 2024). Selected herbarium specimens were transported to the LW Herbarium, which applied HerbScan equipment for their digitisation. Data were captured directly from the received images and stored on JSTOR Global Plants (ITHAKA 2025). Later, data regarding some vascular plants (i.e. rare, relict and endemic) represented in the flora of the Ukrainian Carpathians were mobilised from the LWS Herbarium specimens and published online through the Global Biodiversity Information Facility (GBIF 2025). Depending on available financial sources, different photographic and scanning solutions were applied to produce still images of the herbarium specimens. Since 2021, the digitisation at the LWS Herbarium has become an integral and complex process, including data mobilisation accompanied by specimens imaging, data enhancement and quality control, archiving and publishing. As a result of this effort, recently ca. 8,000 specimens, stored at LWS, were digitised and published on GBIF (Novikov and Sup-Novikova 2023, Novikov et al. 2024). Here, we want to share our experience in organising the digitisation process, which could be helpful for institutions holding small- and medium-sized herbaria and having limited financial support.

## Digitisation workflow

Digitisation is a complex and multi-level process that is organised differently in different institutions, depending on financial capabilities, technical support, qualified personnel, collection size and time frames. Usually, the complete digitisation cycle includes several key stages: *specimen selection* (optional), *pre-digitisation curation* (cleaning, mounting, barcode placing etc.), *databasing, data enhancement* (e.g. georeferencing; optional), *imaging, publishing* and *archiving* (Takano et al. 2019, Nieva de la Hidalga et al. 2020, Thompson and Birch 2023). Haston et al. (2012a) subdivided the general digitisation workflow on three logical lineages (i.e. specimen, data and image workflows) corresponding to pre-digitisation curation, databasing and imaging, respectively. Archiving and publishing are stand-alone processes, but they nevertheless play a crucial role in completing the digitisation workflow (Heberling 2022, De Smedt et al. 2024). Besides this, data cleaning and quality control occur continuously at different stages and/or after some checkpoints (e.g. after each stage or a certain amount of digitised material), improving the resulting data and images, as well as allowing the improvement of the digitisation processes where needed (Nieva de la Hidalga et al. 2020, Engledow 2022, Thompson and Birch 2023, De Smedt et al. 2024).

Different digitisation workflows and their stages can be separated both in time and space and have irregular or linear order, determining two principal digitisation approaches: 'object-to-image-to-data workflow' and 'object-to-data-to-image workflow' (Nelson et al. 2015).

The 'object-to-image-to-data workflow' assumes a linear organisation of digitisation when the specimen operation, imaging and data processing occur successively. It is most widely applied and focuses on obtaining the maximum number of digital images within a limited time. In such a case, data are extracted directly from the specimen image and then processed. Due to high automatisation (sometimes with conveyor belt), often this approach implements fast extraction of a minimum amount of data about the specimen (e.g. barcode and/or specimen ID, taxon name, country and/or local region and, occasionally, collector name and collection date) with the idea that these data can be supplemented or corrected later (Haston et al. 2012a, Haston et al. 2012b, Drinkwater et al. 2014, Nelson et al. 2015, Thompson and Birch 2023, De Smedt et al. 2024). This concept of minimal data providing fits well the standard for Minimum Information about a Digital Specimen (MIDS levels 1 or 2 - Haston et al. (2022), Haston and Chapman (2024), De Smedt et al. (2024)). Minimising required data and limiting it to standard values that can be automatically read by machine (e.g. using optical character recognition technology or barcode scanners) or non-specialists (e.g. volunteers or technicians not qualified in plant biology and regional geography) is a strategy that results in an initial lower number of mistakes. The linear approach, therefore, allows the massive involvement of non-botanists in individual stages, accelerating the general digitisation process. Sometimes, such digitisation is realised by third-party companies specialising in imaging and processing data from natural history collections (e.g. Picturae - NL Digital (2025)).

The linear digitisation approach is probably the most cost-effective, but it also has its drawbacks. Automatisation of the processes and the involvement of unqualified personnel can lead to mistakes, which can potentially remain uncorrected or incomplete for an indefinite time. The second disadvantage of such an approach is the potential delay in obtaining even minimal data since they are obtained only after the production of digital images. In the case of using automatic text recognition technologies (i.e. optical character recognition - OCR or handwritten text recognition - HTR), such a delay may be insignificant (Granzow-de la Cerda and Beach 2010, Drinkwater et al. 2014). Recent progress in data extraction from the herbarium labels using machine-learning technologies is also promising (Takano et al. 2024). However, sometimes automatic text recognition technologies cannot be applied effectively due to insufficient expertise and/or limited sources. Then, a delay in obtaining correct data may occur in the case of barely readable labels, labels with a combination of different handwritings and/or diverse languages and encrypted or abbreviated labels.

At the LWS Herbarium, we applied the second digitisation approach, the 'object-to-data-to-image workflow' (Nelson et al. 2015) also known as a lockstep-process (Lampe and Striebing 2005). It has a more complex organisation of digitisation processes and involves data mobilisation before specimen imaging. It means that labels are first quickly photographed and used to gather the data, which are further verified, modified and supplemented. The final images of the specimens are produced only after databasing. The cascade approach can be helpful when early preselection of the specimens is required and cannot be implemented automatically (e.g. selection of vouchers of some taxa from a specific region). It is also useful in cases where the herbarium labels are barely readable, partly destroyed or covered by the specimens. Such an approach allows critical processing of the mobilised data and placing additional notes on the specimens before imaging. Specimens preselection and preliminary data processing by specialists result in several advantages, particularly obtaining more complete data of better quality (Tann and Flemons 2008, Granzow-de la Cerda and Beach 2010), albeit for a smaller number of specimens. These enhanced data of MIDS Levels 2 and 3 (Haston and Chapman 2024) can be used in investigations and published without waiting for the production of digital images. The separation and independence of stages allow optimisation of the digitisation process (Tann and Flemons 2008, Granzow-de la Cerda and Beach 2010). Such an approach also justifies itself in the case of batch digitisation, when digitisation is conducted unsystematically, with lengthy interruptions and when the staff and available equipment may change significantly. In the cascade approach, the physical specimen is directly involved twice: once during the label imaging and once during the final specimen imaging. This results in an extra contact with the specimen and, consequently, extra step of quality control that allows us to catch mistakes which are invisible or overlooked on the images. Extra contact with the original specimen also allows additional specimen selection and solving other issues (e.g. incorrect placing or mounted labels) if such issues are discovered. Instead, the linear approach commonly means only one direct contact event with the original specimen, as a result of which its digital image is received. Such a digital image serves as a so-called 'digital specimen' (Webster 2018, Lannom et al. 2020), which is used for all further operations (i.e. data mobilisation and data enhancement). However, if there is no strict need to pre-select and additionally filter the specimens (e.g. belonging to certain taxa from a particular region) before imaging, the application of a cascade approach can be unjustified as it results in extra load on the collection during the specimens' handling.

The main disadvantage of the cascade approach is its complexity. Requiring qualified personnel makes digitisation more labour-intensive, expensive and time-consuming (Tann and Flemons 2008, Mononen et al. 2014, Brenskelle et al. 2020). In the cascade approach, the data interpretation and processing must be implemented by experts. In particular, our experience showed that specimens georeferencing by unqualified staff led to misidentified locations that are hard to detect without checking the initial locality data. Amongst other critical mistakes were incorrectly indicated IDs and arbitrary and incorrectly interpreted specific data (e.g. abbreviations, collector names and dates), which also required checking the original material. Minor mistakes included overlooked specimens, misspellings of names of taxa and incomplete or partly missing data (Novikov 2024). In contrast to the 'capture minimal data' strategy applied in the linear approach, the cascade approach implements a 'data-intensive' strategy (Granzow-de la Cerda and Beach 2010), with data quality prioritised and specialists involved as much as possible. In the linear approach, data often initially corresponds only to MIDS levels 1 or 2 (sometimes partially) and later can reach a higher level or remain the same. In contrast, in a cascade approach, obtained data initially corresponds to MIDS level 2 or, often, even meets the criteria of the MIDS level 3.

Current digitisation at the LWS Herbarium is focused on the priority group comprising specimens of endemic, rare and relict taxa of the Ukrainian Carpathians (Novikov et al. 2024b). Hence, due to strict taxonomic and geographic limitations, there is a need to preselect the specimens before their digitisation. In some cases, georeferencing is crucial to verify whether the specimens fit the target group. On the administrative level, it was also decided to keep the old identification system (six-digit accession numbers serving as locally unique identifiers - LUIDs) to mark the specimens within the collection. In addition, globally unique identifiers (GUIDs) in the form of universally unique identifiers (UUIDs) were assigned to the specimen records in the dataset. The GUIDs serve for data publishing purposes only (e.g. in GBIF) and, at the moment, are not displayed on the herbarium sheets in any way. After developing our own virtual herbarium, it is planned to use GUIDs to generate the permalinks and place QR-codes on the herbarium sheets with the corresponding specimens. Preselection with preliminary analysis of the label data allows us to prepare an initial list of specimens and place the barcodes with specimen IDs on them before their digitisation. This approach also allowed us to update the existing taxonomy in our collection, merge the specimens placed under different synonymic names into a single folder and place notae criticae with recent identifications on the specimens before their imaging. Therefore, it was decided to follow the cascade digitisation approach using the list of tested equipment and services (Suppl. material 1).

### Stage 1. Imaging the labels

Ideally, for the digitisation of an entire herbarium, the labels for all specimens should be captured, folder by folder and processed respectively. At the same time, all new incoming specimens should also be digitised before entering the collection. However, in the case of financial issues and staff rotations, choosing the specimens prioritised for digitisation could be a more viable strategy, allowing us to digitise small portions of the collection case by case. This requires extra physical handling of the specimens, but it avoids producing an extra amount of files that can remain unprocessed for years or become outdated (e.g. if the specimen has been re-identified).

**Figure 1.**
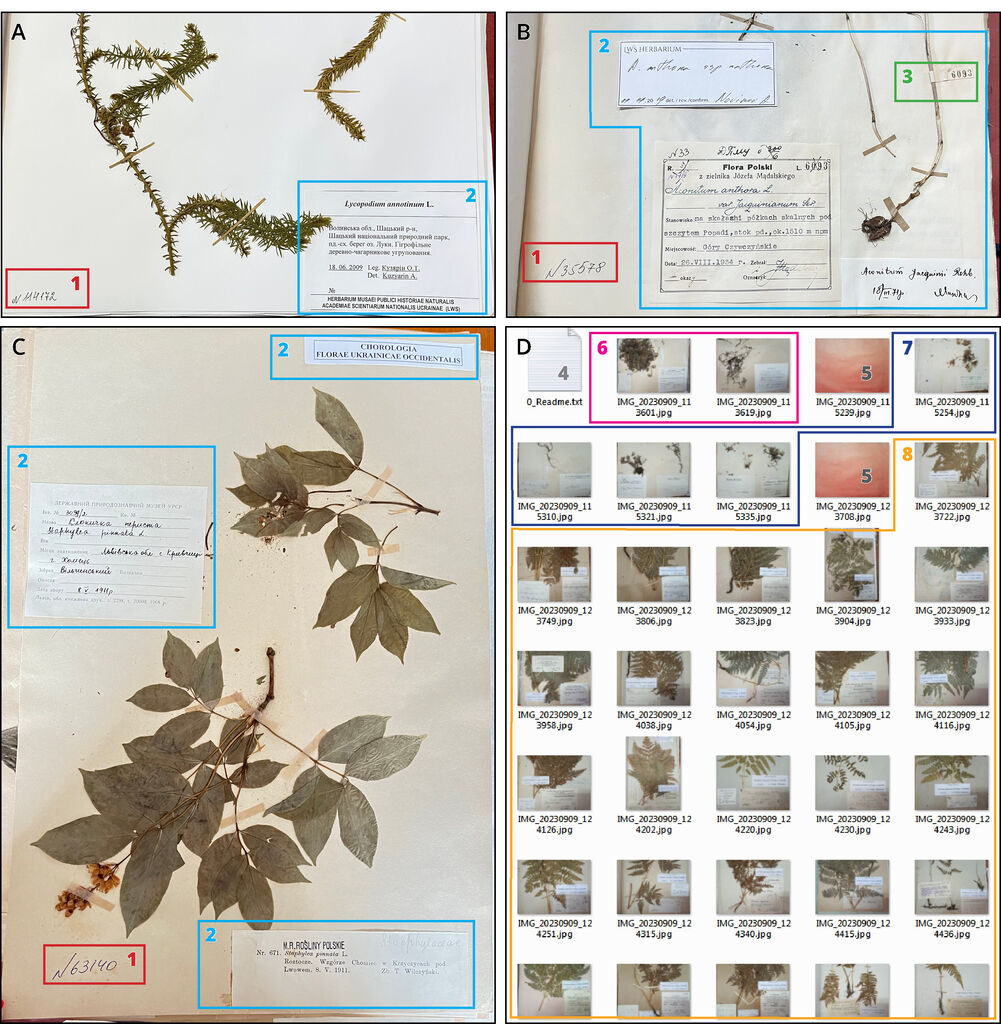
The common view and organisation of the pictures captured during the labels imaging. Examples of horizontal (A, B) and vertical (C) images containing elements of the herbarium sheet required for the databasing: 1 - accession number; 2 - label(s); 3 - field number. Example of the files organisation (D) representing the key elements of the folder with labels images: 4 - readme file with a list of species/infraspecies represented in the current folder and supporting notes; 5 - images of empty table captured after the last specimen of each species/infraspecies helping to navigate between them quickly; 6 - the label images of the first species/infraspecies; 7 - the label images of the second species/infraspecies; 8 - the label images of the third species/infraspecies.

**Figure 2.**
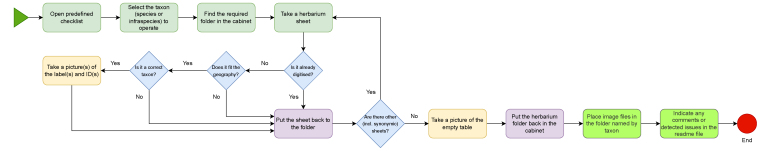
The flow chart of imaging the labels of specimens of predefined taxa from a particular region. The herbarium folders are precisely checked for the specimens corresponding to the predefined checklist. All potential synonyms must be checked and respective notes must be made in a checklist and/or readme file. The labels and IDs of all specimens meeting the checklist criteria and doubtful ones (requiring additional examination and/or clarification of the collection region) are photographed. All the specimens that do not meet the predefined checklist are omitted. There is no need to make extra-detailed (excepting doubtful specimens) photos at this stage, but the quality of the resulting picture must be enough to recognise the text. The same illumination and orientation of the pictures are preferable, as it later helps to process the images faster.

Volunteers or technical staff with moderate experience in the taxonomy and geography of the region can be involved. The labels and IDs can be photographed using any digital camera or smartphone. In the LWS Herbarium, labels are usually located in the bottom right corner and accession numbers (six-digit IDs, with sometimes omitted zeros at the beginning) are in the bottom left corner of the herbarium sheet. Therefore, only the bottom part of the herbarium sheet must be photographed (Fig. 1). All pictures must have the same horizontal orientation and size - this will save time during their processing as there will be no need to scale and/or rotate them. However, it sometimes happens that labels are located in the upper part of the herbarium sheet. In such a case, the picture of the entire sheet is taken. The same herbarium can store specimens of the same taxon under different names, as accepted names and taxonomic vision have changed over time. Therefore, it is essential to prepare and follow the checklist of predefined taxa and check both the accepted name and all their possible synonyms (Fig. 2).

The image files should preferably be sorted into folders named correspondingly after the processed taxa (species or infraspecies). However, in the case of intensive digitisation with many different taxa processed daily, placing the files in folders named by working date could be more convenient, with the prospect sorting the files by taxa later or without such sorting at all.

During the bulk photographing of the specimens' labels in the LWS Herbarium, the contrasting pictures (e.g. of an empty table or blank sheet) are made after the last specimen of each species or infraspecies (Fig. 1D). Such contrasting pictures help to quickly navigate through between the image portions and preliminarily estimate the number of pictures for each taxon. Each folder with the multiple pictures obtained in one day must be additionally supported by a text readme file containing the list of processed taxa and any comments on discovered issues.

### Stage 2. Data mobilisation and processing

The LWS Herbarium contains ca. 147,000 specimens, many of which were collected in the second half of the 1800s and in the 1900s by a few dozen principal collectors. These specimens mostly have handwritten labels, some of which are partly damaged. They were primarily written in Polish, Ukrainian and Russian. However, many labels here were still written in Latin, Slovakian, Romanian, French, German and Hungarian. Few recent specimens were annotated in English. Some specimens combine labels in several languages. Therefore, processing of such specimens requires at least basic skills in multiple languages and palaeographics.

**Figure 3.**
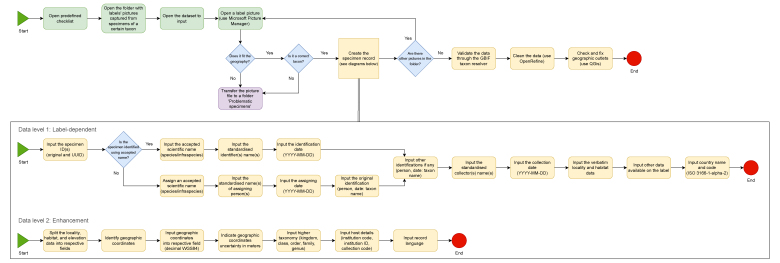
The flow chart of data mobilisation from the herbarium labels and data enhancement. Initially, the data are stored in a Microsoft Excel table. This table is formatted following the DarwinCore standard (TDWG 2025) with the idea to conform to the GBIF Occurence class of the datasets (GBIF 2025b). Preliminary data cleaning is performed in the native environment of Microsoft Excel after each portion of work (e.g. after finishing processing all taxa from one family or at the end of the year). However, such data cleaning is not enough for further data processing. Therefore, advanced data cleaning is realised using OpenRefine software (OpenRefine Community 2025). It is worth noting that, before uploading to GBIF, the data must be validated in consistency with the GBIF backbone taxonomy (GBIF Secretariat 2023). Three other principal plant checklists, i.e. World Flora Online (WFO 2025a), Plants of the World (POWO 2025) and World Plants (WP - Hassler 2025) also have to be taken into consideration. To validate the taxonomy in consistency with WFO, the Name Matching Tool (WFO 2025b) can be applied. Mistakes in georeferencing can be checked in different ways, for example, using QGIS (QGIS Association 2025) or similar software after completing the dataset.

The data from the labels at the LWS Herbarium are extracted only by qualified persons directly from the herbarium labels using the protocol depicted in Fig. 3. The person working with primary data must be familiar with the taxonomy of the selected plant groups and the regional geography. Further data cleaning and cross-validation should preferably be realised by another person experienced in general biodiversity data processing. Otherwise, engaging volunteers requires the development of an advanced crowd-sourcing platform with a rating system, as was proposed by Santos et al. (2020). In such a case, different permission and trust levels must be acquired depending on the transcribers' performance.

The LWS Herbarium does not use OCR or HTR technologies to parse the data from the herbarium labels due to lack of expertise and the significant variation of handwriting and languages presented on labels. Unfortunately, the test application of Transkribus (READ-COOP SCE 2024) showed insufficient results and we failed to train it. At the same time, the relatively low number of specimens (i.e. 50-100 specimens per day) can be processed by hand. Therefore, we decided to use the traditional data extraction method until we gain expertise in this field. Besides this, new technologies implementing artificial intelligence in data parsing and enhancement (e.g. DiSScover - DiSSCo (2025a) and Hespi - Turnbull et al. (2024)) are actively developed. Probably, in the near future, data extraction from the herbarium labels will be released automatically with high accuracy.

However, we found it helpful for data processing to create an additional list of the collectors (at the moment, it comprises 374 records), which contains standardised collectors' names in English and shortenings following the IPNI database (IPNI 2025), alternative names (e.g. in original language and other languages), years of life, years of research activity, research interests and geographic coverage. There are also provided links to respective biographic data on IPNI (IPNI 2025), Wikidata (Wikimedia Foundation 2025), HUH Index of Botanists (Harvard University Herbaria & Libraries 2013), Bionomia (Shorthouse 2025), VIAF (OCLC 2025) and other online sources if available. The list is supported by photos of respective handwriting serving as a comparison benchmark. Applying standardised names of collectors allows better data navigation, filtering and sorting. Moreover, there is no need each time to type the correct name or verify its correct spelling, especially if it applies diacritic marks. This saves a considerable amount of time.

### Stage 3. Pre-imaging preparation of the specimens

**Figure 4.**
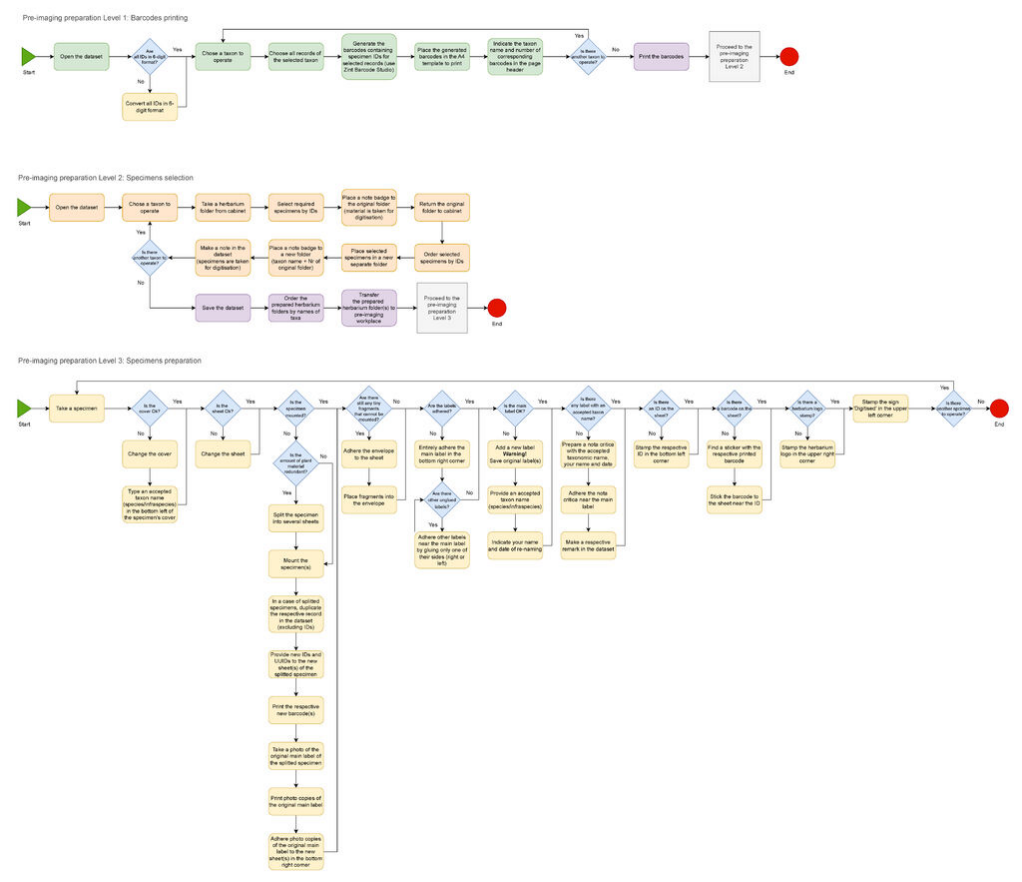
The flow chart of pre-imaging preparation of the specimens. Based on the dataset developed during the label transcription, required specimens are selected from the collection and placed in a separate working table for further pre-imaging preparation. The initial preparation of the specimen before imaging includes mounting unbound parts of the specimen and labels to the herbarium sheet, packing the small plant parts into the envelope attached to the sheet, restoring labels or preparing new ones and changing damaged herbarium sheets and/or covers. The second part of the specimen preparation includes placing the stamp of the herbarium, checking and stamping (if absent) the ID (specimen accession number), attaching the barcode label, attaching the nota critica with re-identification (if needed) and stamping the sign 'Digitised'. The barcode can be potentially detached, so the stamping or writing the accession number directly on the herbarium sheet is required. If the specimen is missing or has a duplicate accession number, the new accession number is designated to it and indicated in the dataset. At the LWS Herbarium, the barcodes with six-digit accession numbers as locally unique IDs (LUIDs) are applied. Such barcodes are prepared using Zint Barcode Studio software (Stuart 2024), based on the initial dataset before the imaging. They are laser-printed in high-quality mode on the stickers (38.1 mm × 21.2 mm) and adhered to the specimens before their imaging. The application of additional QR codes with GUIDs will be considered for the LWS Herbarium when the common digitisation strategy for all the collections kept at the State Museum of Natural History of the NAS of Ukraine is agreed upon.

Pre-imaging preparation of the specimens is a multi-level process that includes different tasks, most of which can be done by technicians, but specialists must still be involved in quality control. Our experience revealed that, even after qualified transcription of the labels, ca. 5-7% of specimens require additional data modifications and clarifications at the stage of pre-imaging preparation (Fig. 4).

Nieva de la Hidalga et al. (2020) pointed out that each image of the herbarium specimen should contain five principal image elements required for proper image processing and quality control: colour reference chart, scale bar, label, barcode and institution name. Besides this, we have found it helpful to mark already digitised specimens to avoid duplicate processing. Special stickers or a stamp 'Digitised' can be applied for this purpose. Preparation and mounting the stickers is very time-consuming, but it benefits from the possibility of removing them in case of need.

Colour reference charts (colour checkers) are generally required for digitisation, including the herbarium specimens digitisation (van Dormolen 2012, JSTOR 2018, Guiraud et al. 2019, Rieger et al. 2023). They ensure the proper adjustment of the colours and white balance in the images. Amongst the most popular colour reference charts for herbarium digitisation are X-Rite ColorChecker Classic Nano (40 mm × 24 mm) and ISA Golden Thread Object-Level Target x1 (235 mm × 25 mm). There are many other options (e.g. Kodak/Tiffen Q13 consisting of two pieces, 203 mm × 60 mm each), which differ widely by the number of colour patches, their organisation (linear or tabular), size and price. When choosing the colour reference chart, it is important to consider two principal aspects: its size and the ability to automatically calibrate it with specialised software (e.g. ImageZebra (2025)). If the chart is too large, it will overlap the specimen. The linear charts can be placed beside the specimen, along its margin. However, the tabular charts are usually quite wide. Therefore, they must be placed directly over the herbarium sheet to avoid the appearance of extensive empty space around the sheet on the image. If the chart is not allowed for automatic processing, extra time will be required to calibrate the images. Application of outdated or low-quality charts can result in incorrect image calibration. For the same reason, applying charts not widely used requires an additional indication of its producer and type. The best option in such a case is to indicate the reference colour values for all patches of the applied chart in CIELAB (L*a*b*) format (Carter et al. 2019). Otherwise, they can be confused and images calibrated improperly. Our previous studies (Novikov et al. 2024b) showed that the ISA Golden Thread Object-Level Target is the best choice since it is less affected by colour degradation. This target is also preferable due to the extended set of grey patches and the presence of an additional resolution test pattern (so called 'convergent line pair gauge', CLP) for evaluating the images' geometric distortion and spatial frequency response.

Scale bar, crucial for estimation of the sizes of the specimen, is present on many colour reference charts. However, many herbaria use branded scale bars placed beside the colour reference chart. Such scale bars can be printed on special transparent non-reflective film (e.g. biaxially-orientated polyethylene terephthalate (BoPET) film DuPont Mylar), which allows them to be placed over the specimen. At the LWS Herbarium, the X-Rite ColorChecker Classic Mini and ISA Golden Thread Object-Level Target charts, each containing scale bars, are used for digitising specimens of vascular plants. Therefore, we do not apply an additional branded scale bar since it requires additional space and manipulations. However, for digitising specimens of non-vascular plants, we use a smaller colour reference chart, Charttu Nano (without a preprinted scale bar), supplemented by an additional branded scale bar.

Herbarium digitisation can require introducing a new specimen ID system, which can either be used in parallel with the existing one or to replace the latter. However, replacing old IDs with new ones can create errors when specimens have already been published and cited in publications with old IDs. Therefore, the new ID system must have unique IDs that do not overlap with the old ones. This can be realised by providing a unique prefix to the new IDs or by applying GUIDs. The image must contain a machine-readable barcode or QR code for better automatisation. Sometimes, specimen IDs can be mistakenly duplicated or missing in the collection. The preselection of specimens, printing and placing the barcodes (Code 128, ISO 15417) or QR codes (ISO 18004) corresponding to specimens' original IDs, as well as identification of erroneous IDs, are time-consuming. Therefore, a new ID system is often introduced when barcoded or QR-coded IDs are printed and placed consequently or randomly on the specimens. A good practice in such a case is using globally unique identifiers (GUIDs) to ensure effective digital data curation and databasing (James et al. 2018, Nelson et al. 2018, Kirchhoff et al. 2018).

**Figure 5.**
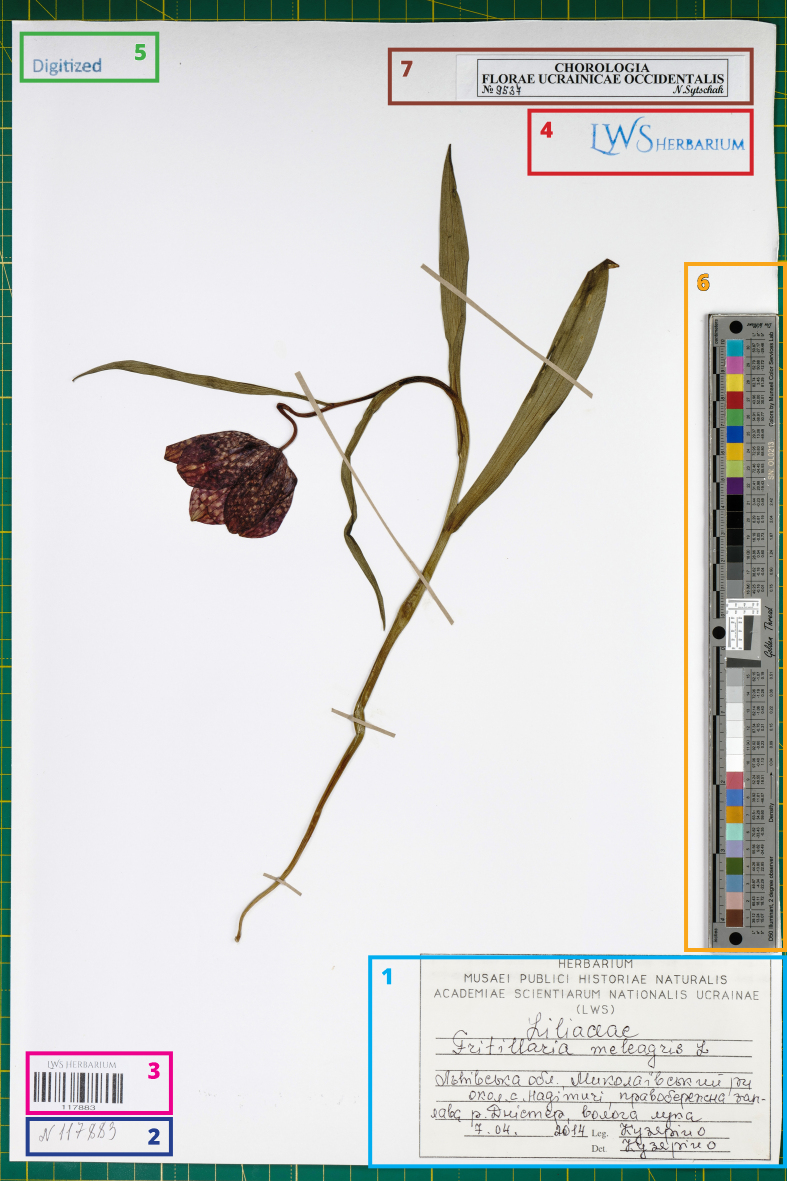
The final image of the entire specimen. The key elements represented on the herbarium sheet during the imaging: 1 - principal label; 2 - handwritten or stamped accession number (LUID); 3 - barcode corresponding to the accession number; 4 - herbarium stamp; 5 - stamp confirming the digitisation of the specimen; 6 - colour reference chart (ISA Golden Thread object-level target); 7 - supplementary label (indicating inclusion of the specimen in a local research programme).

Keeping the same order of the elements on the herbarium sheet is useful. It helps to navigate over the specimen elements on its image. At the LWS Herbarium, the specimen label is typically placed in the right bottom corner, while the specimen accession number (LUID) is written or typed in the left bottom corner (Fig. 5). The barcode corresponding to the specimen accession number is attached just near or slightly over it. The stamp of the herbarium is placed in the right upper corner or, if not possible, in the upper centre of the herbarium sheet. Just before imaging the specimen, the stamp 'Digitised' is placed in the upper left corner of the sheet (such stamp can be also placed after imaging the specimen). Notae criticae, if present and possible, are attached near the original label in the right part of the sheet. The colour reference chart, if possible, is placed on the right side of the herbarium sheet just before imaging.

### Stage 4. Imaging the specimens

**Figure 6.**
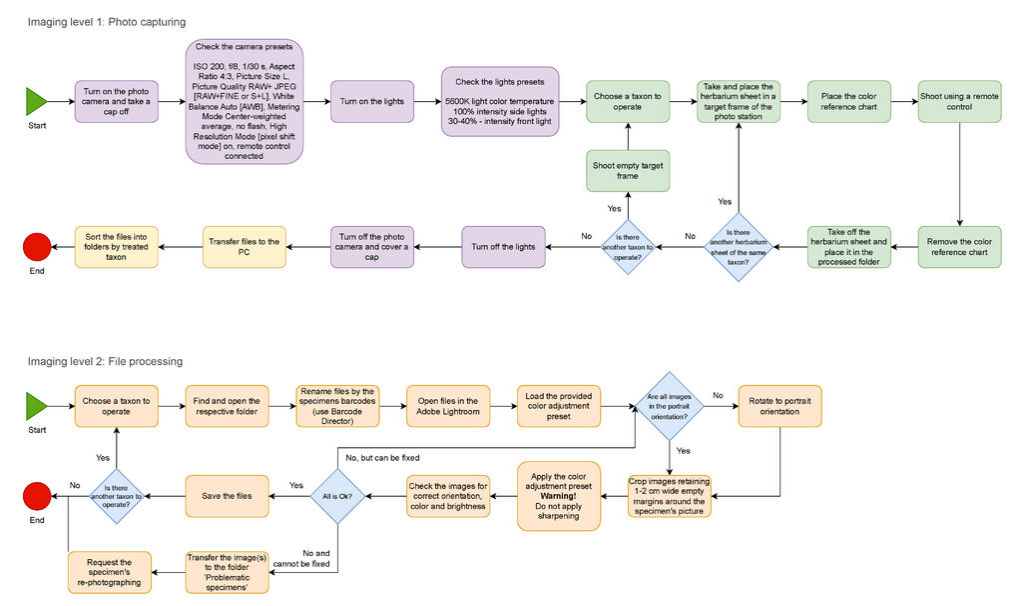
The flow chart of imaging and image processing. Before imaging, attention is paid to several important steps. In particular, the presence of all key elements i.e. principal label, ID, barcode, herbarium stamp, digitisation stamp, colour reference chart and supplementary labels must be checked on the herbarium sheet. The illumination must be adjusted to indicate preferences and its uniformity along the specimen must be checked. The camera lens must be kept clean. In case of dirt or dust, it must be carefully removed with the specially provided tools (brush and microfibre napkin). The free space availability on the SD card and predefined camera preferences are also checked. The herbarium specimens must be handled carefully, one by one, avoiding mixing and damaging. During the processing of the images, it is important to keep RAW files intact; only renaming is allowed. The images in distribution format (i.e. JPEG) are the objects to further manipulations (i.e. cropping, rotation, colour adjustment etc.). The sharpening of images is not permitted.

After pre-imaging preparation, the imaging can be done by technicians, volunteers or outsourced to a company. This stage is entirely related to image production, adjustment and file organisation (Fig. 6) and almost eliminates the risk of scientific-related mistakes.

The final imaging of the herbarium specimens can be realised using different technical solutions, i.e. scanners (including flatbed and planetary - see JSTOR (2018), Roma-Marzio et al. (2023), Tasenkevich et al. (2024) for examples) and photo cameras (including DSLR and mirrorless - see Thiers et al. (2016), Sweeney et al. (2018), Takano et al. (2019), Davis et al. (2021) and DiSSCo 2025b for examples). Each solution has its pros and cons. For example, scanners are relatively expensive, slow and have a shorter lifespan. Applying flatbed scanners requires additional constructions to revert it and place the specimen under the scanning surface. Such construction is usually bulky (e.g. HerbScan), requiring extra space to hold and providing instead less space to manipulate the specimen. Moreover, the scanner should not contact directly with the specimen to avoid damage to the specimen and pollution of the scanning surface. Master files received with scanners are generally saved in TIFF format and can reach 700 Mb or more, which requires superior storage capacities. However, scanners with CCD sensors often produce images of better quality (especially regarding the sharpness and colour reproduction) and resolution (Waltham 2013, Mehta et al. 2015, Tejas et al. 2022) compared to the commonly used photocameras with CMOS sensors. Photo stations are usually less expensive to construct, modular and better suited to various working spaces. A distantly mounted photo camera provides much more space to manipulate the specimen. Photo cameras produce images faster and can be set to automatically create the master files (generally in RAW format, but sometimes also in TIFF) and distribution files (lossy images in JPEG format). The obtained files are comparatively smaller in size and require fewer saving capacities. However, the quality of obtained images strongly depends on the camera and lens quality as well as on illumination conditions. Resulting images can have different optical distortions and uneven lightness uniformity.

The quality is crucial for the images of herbarium specimens, as they can be used for investigations at different magnifications. Nieva de la Hidalga et al. (2020) ascertained general requirements for the images of the herbarium sheets (both stored and web-distributed) and pointed out that colour reproduction accuracy (ΔE) should not exceed 5 points. Such colour reproduction accuracy conforms to the two-star quality level of the current FADGI guide (Rieger et al. 2023). Our previous studies (Novikov et al. 2023) showed that many herbarium specimens' images provided in the virtual herbaria do not meet this criterion, reaching only a one-star quality level. Most plants lose their natural colours during herbarisation and continue to change their colour over time during storage. Considering this, the value of accurate colour reproduction is questionable. Nieva de la Hidalga et al. (2020) also suggested 72 PPI image resolution sufficient for web publishing and a resolution of 600 PPI - as suitable for preservation and research. As the authors pointed out, such estimates are fair for the scanned images, but hardly convertible to apply in photography. Based on our experience, we believe that 10 Mp (ca. 72-96 PPI) is the minimum resolution of photo-captured images of the herbarium specimens for web publishing and 20 Mp is the minimum limit for research and long-term storage.

Nieva de la Hidalga et al. (2020) suggested three types of files that should be produced as a result of the herbarium digitisation, i.e. archiving master file (TIFF), hi-res production file (JPEG2000) and lossy distribution image (JPEG). At the LWS Herbarium, we apply only two types: archiving master file (RAW) and distribution file (JPEG). The hi-res production files (80 Mp maximum) can be generated later from the master files if needed. Moreover, we provide distribution files in the highest possible resolution (i.e. 16 or 40 Mp), which is enough for most production and research purposes. Acceptance of such a strategy saves time since both file types are automatically generated by the photo camera.

It is worth noting that image sharpening is not allowed for the archiving master files (van Dormolen 2012, Rieger et al. 2023). It is also recommended to avoid sharpening adjustments in the distribution files. The general processing of the files must include only fixing the image orientation (in most cases to portrait), cropping (FADGI recommends leaving some free space around the specimen; Rieger et al. (2023)) and colour and white balance adjustment (using presets generated for the applied colour reference chart following the producer's recommendations).

### Stage 5. Final verification and publishing

**Figure 7.**
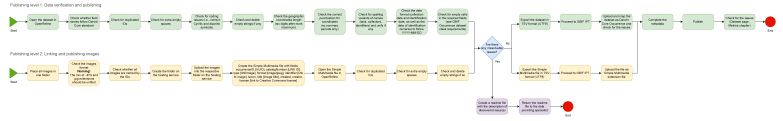
The flow chart of final data verification, cross-linking with images and publishing on GBIF. Special attention is paid to data uniformity and consistency. Data from different rows must not be mixed. Data verification is realised in the OpenRefine (OpenRefine Community 2025) environment following the protocol illustrated on this flow chart. It is also important to regularly check and keep the UTF-8 coding format of the dataset. Data are published to GBIF using the IPT of the State Museum of the Natural History of the NAS of Ukraine. The multimedia files are deposited to the NIRD Service Platform (Sigma2 2025) and linked to the GBIF dataset using the Simple Multimedia extension file. The structure and data represented in the Simple Multimedia extension file are additionally verified in OpenRefine before uploading to GBIF.

The publishing stage includes publishing the data, publishing the images and crosslinking the data and images (Fig. 7). It also involves data transformation to meet the standard applied in the Data Centre Biodiversity of Ukraine (DCBU 2025), as it does not follow DarwinCore. Such data transformation is a sophisticated process mostly manually implemented by the service provider or trained technicians. Therefore, it is not described here. In general, final data verification and publishing can be done by technician staff or volunteers with elementary experience in IT.

Biodiversity data should be published and permanently archived in appropriate, trusted, general or domain-specific repositories (Egloff et al. 2016) and follow the FAIR and FAIR Digital Objects (FDO) principles (Wilkinson et al. 2016, Guizzardi and Sales 2022, GoFAIR 2025). Amongst the important requirements for published data are their persistence, the ability to clearly identify them on the web (e.g. using DOI) and the ability to track the changes. In such a sense, GBIF (2025a) seems to be one of the best solutions for data publishing. However, it is not a specialised virtual herbarium like JACQ (JACQ consortium 2025), Open Herbarium (Open Herbarium 2025) or Reflora (Institute of Research Rio de Janeiro Botanical Garden 2025). GBIF serves as a global data aggregator with outperformed functionality. At the same time, virtual herbaria are primary data providers with their specific functional peculiarities mainly focused on work with images and extended specimen annotations.

The LWS Herbarium does not have its virtual herbarium platform and Ukraine does not have a joint specialised national platform or portal for publishing data on digitised collections. Only two independent Ukrainian platforms allow the publication of biodiversity data, including those obtained from the digitised collections, i.e. the Ukrainian Biodiversity Information Network (UkrBin 2025) and the Data Centre Biodiversity of Ukraine (DCBU 2025). Both platforms publish different kinds of biodiversity data, including living observations, published reports and data mobilised from natural history collections. However, both platforms are self-running and do not exchange data with GBIF, limiting data integration and visibility. Moreover, they do not yet allow bulk data export, which makes data extraction laborious. As a result, data published through these platforms are weakly integrated into the research compared to GBIF (2025a).

Considering this, the LWS Herbarium data are published online on several platforms simultaneously, i.e. GBIF (2025a), Open Herbarium (2025) and DCBU (2025). GBIF is a principal data-providing platform where the data are deposited as Occurrence class datasets through the Integrated Publishing Toolkit (IPT - Robertson et al. (2014), GBIF (2025c)). The images are hosted on the NIRD Service Platform (Sigma2 2025), kindly provided by the Norwegian GBIF node and synchronised with the dataset using Simple Multimedia extension files (Robertson et al. 2014). The Open Herbarium is a free online platform for publishing virtual herbarium collections developed by Arizona State University (ASU) on the basis of the Symbiota software (Gries et al. 2014, Symbiota Support Hub 2025). The images of the digitised LWS specimens are stored on kindly provided ASU servers and synchronised with the Open Herbarium platform. The DCBU serves as the internal platform to publish and host digitised materials, running independently by the State Museum of Natural History of the NAS of Ukraine. Images of the LWS specimens in the highest possible resolution can be accessed through GBIF or Open Herbarium. At the same time, due to technical limitations, the DCBU hosts images of reduced quality (2.25 Mp maximum). These platforms are updated manually and irregularly, without the application of any collection management system.

### Stage 6. Archiving

In archiving, the application of storage media (e.g. optical discs) produced by different manufacturers is advisable to avoid possible manufacturing defects and potentially low production quality (Rieger et al. 2023). When archiving, at least three backup copies must be created using at least two storage media and one of the copies must be stored outside the originating institution (so called '3-2-1 rule' - Perkel (2019)).

Similarly to the case of data publishing, the stored materials must be well-structured, well-annotated, clearly identifiable and represented in open and raw formats (Hart et al. 2016, Wilkinson et al. 2016). There are no clear terms of how long the digitised materials should be preserved, but at least a few decades are expected. Appropriate archiving directly designates the data lifespan. Therefore, it must be realised by archive specialists and cannot be delegated to volunteers or inexperienced staff. De Smedt et al. (2024) recommend cold storage (i.e. archiving without manipulating the files after that) of herbarium images daily. Similarly to the Herbarium of the Meise Botanic Garden (BR), the images at the LWS Herbarium are archived after renaming, processing and quality control. However, at the LWS Herbarium, the images are archived only at the end of the digitisation bench (once per several months). They are stored together with metadata and dataset files describing these images.

**Figure 8.**
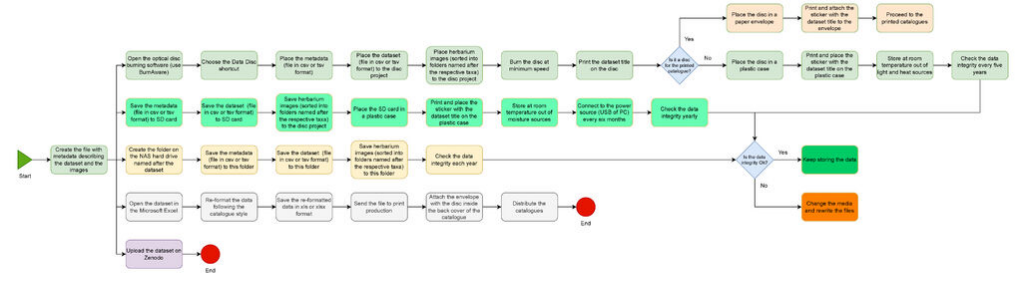
The flow chart of the data and images archiving. At the LWS Herbarium, the data are archived on three types of the storage media: (a) on the internal server using the service of the National Academy of Sciences of Ukraine; (b) on the SD memory cards (128 GB SanDisk Extreme Pro cards, which can store ca. 1,024 image files in RW2 format or 6,400 in JPEG format); (c) on the BluRay MABL discs (25 GB Verbatim SL BD-R, which can store ca. 200 image files in RW2 format or 1,250 in JPEG format). Such a combination of storage media (magnetic, electronic and optical) provides long-term preservation of the digitised data and images. Besides this, datasets are also archived on Zenodo (CERN 2025). Since 2024, the data regarding the digitised specimens have been published as printed herbarium catalogues (e.g. Novikov et al. 2024a, Novikov et al. 2024b), stored also on Zenodo (CERN 2025). These catalogues are supported by DVDs (Verbatim Archival Grade Gold DVD-R) with datasets and images in JPEG format. The catalogues with the attached discs are distributed through the libraries that serve as additional archiving agents (Harvey and Mahard 2020, Frick and Greeff 2021).

Simultaneous use of several different types of storage media (e.g. magnetic tapes and external hard drives) for the herbarium digitisation is advised by Haston et al. (2012b). Application of storage produced by different manufacturers is also advisable to avoid possible manufacturing defects and potentially low production quality (Rieger et al. 2023). Following these recommendations, in the LWS Herbarium, the so-called '3-2-1 archiving rule' is applied (Perkel 2019). This means that at least three backup copies are stored on at least two different types of storage media and at least one copy is stored outside the LWS Herbarium (Fig. 8).

However, combination of several different archiving media has drawbacks, namely, controlling the preservation conditions and data persistence can be complicated. Moreover, the State Museum of Natural History of the NAS of Ukraine has no common archiving and data management strategy yet. Developing a common archiving strategy would help resolve questions about file formats, priority long-term storage media, cyclic media renewal terms and the distribution of personnel roles in data archiving.

## Conclusions

The digitisation of the herbarium specimens at the LWS Herbarium is a multilevel process organised in a cascade manner. This so-called 'object-to-data-to-image' workflow prioritises data extraction and enhancement to meet MIDS levels 2 and 3. Such digitisation is complicated, but allows additional access to physical specimens (once during the label photographing and, secondly, during the capture of the entire specimens), resulting in an extra step of quality control. In contrast, in the case of a linear approach, the physical specimen is usually accessed only once and the later processing is realised using its digital copy (so-called 'digital specimen'). Additional access to the specimens increases the negative load on the collection as a result of extra handling. However, it allows the revealing and early fixing of errors and gaps in data (e.g. incorrect identifications, duplicating numbers, misplaced specimens etc.) that can be overlooked in the case of digital specimen use. At the same time, such an approach allows early preselection of the specimens for digitisation and their extra filtering before the image of the entire specimens. Such filtering allows revealing the specimens that are out of digitisation priority, but were mistakenly considered as such. For example, if the specimen has been considered as collected in the Ukrainian Carpathians, but after detailed explorations, it appeared to be collected out of this geographic range. Hence, the cascade approach principally focuses on thematic (i.e. focused on specific taxa and certain regions) digitisation in parallel to data mobilisation, enhancement and publishing (e.g. through GBIF). Similarly to the linear approach, it allows the involvement of volunteers and non-qualified staff at certain stages. However, data-intensive strategy applied in a cascade approach requires extra involvement of specialists familiar with the plant biology (especially, nomenclature and taxonomy) and regional geography.

The digitisation workflow described in this article and illustrated by the detailed schemas is intended to help other herbaria with limited budgets to effectively organise the digitisation and virtual publishing of the data regarding their collections. Clarifications and notes are provided on each digitisation stage aimed to help choose appropriate equipment, materials, archiving media and other technical solutions.

## Supplementary Material

0274931F-16F7-5715-A404-F7C45A0BFAA9 10.3897/BDJ.13.e148861.suppl1 Supplementary material 1 The equipment, materials, software and other sources applied during the digitisation of the specimens at the LWS Herbarium Data type Textual description Brief description The supplement contains the list of the equipment, materials, software and online resources applied during the digitisation of the specimens at the LWS Herbarium. File: oo_1238291.docx https://binary.pensoft.net/file/1238291 Andriy Novikov & Viktor Nachychko
