# Supplementary material for: The digitisation workflow of the herbarium of the State Museum of Natural History of the NAS of Ukraine (LWS)
Source: Biodivers Data J. 2025 Mar 28;13:e148861. doi: 10.3897/BDJ.13.e148861 (PMC11971642; doi:10.3897/BDJ.13.e148861)
Supplement: Supplementary material 1 — The equipment, materials, software and other sources applied during the digitisation of the specimens at the LWS Herbarium [file bdj-13-e148861-s001.docx]

**The equipment, materials, software and other sources applied during the digitisation of the specimens at the LWS herbarium**

**Main equipment**

**Light:** Two led studio lights Yongnuo YN-300 Air (3200-5600K) in sides and one led studio light Yongnuo YN-600 Air (3200-5600K) *or* MyGear SL-288APC (3200-5600K) in front. Studio lights are preset to 5600K only (3200K set to zero position), since such color temperature is the closest to the day light.

**Tripod and mountings:** Horisontal tripod Beike QZSD Q202A. Two universal mountings MagicArm 7” for the studio lights Yongnuo YN-300 Air. One universal mounting Visico CL-035 *or* Falcon CL-35 for the studio light Yongnuo YN-600 Air

**Photo set variant 1 (applied before 2024):** Photo camera Canon EOS 800D (APS-C, 24 Mp). Lens Tokina AF 35mm f/2.8 AT-X PRO DX Macro. Memory card SanDisck Extreme Pro 128 GB micro SDXC UHS-I. Lens filters absent. Remote control Shoot RS-60E3 *or* Canon Rs 60e3. Two additional batteries (KingMa LP-E17 *or* Canon LP-E17). Two chargers (Canon LC-E17 *and* KingMa LC-E17).

*Camera presets:* Manual shoot mode, no flash, ISO 200, f/5.6, 1/50 s, aspect ratio 4:3, white balance auto (AWB), metering mode center-weighted average, picture size L, picture quality RAW+L (RAW+JPEG).

**Photo set variant 2 (currently applied):** Photo camera Panasonic Lumix DC-G9 (Micro 3/4, 20 (80) Mp). Lens Olympus M.Zuiko Digital ED 30mm f/3.5 Macro. Memory card SanDisck Extreme Pro 128 GB micro SDXC UHS-I. Lens filters absent. Remote control Shoot DMW-RS1 *or* DMW-RSL1, CR-D1. Two additional batteries (Newell DMW-BLK22 *or* Panasonic DMW-BLK22).

*Camera presets:* Manual shoot mode, ISO 200, f/8, 1/30 s, white balance auto (AWB), metering mode center-weighted average, picture size L, High Resolution (pixel shift) mode on, picture quality RAW+Fine (RAW+JPEG).

**Applied color reference charts:** X-Rite ColorChecker Classic Mini (109×64 mm, applied before 2024). ISA Golden Thread Object-Level Target x1 (235×25 mm, includes additional CLP gauge).

**Recommended color reference charts:**

*Mini format charts:* Datacolor SpyderCheckr SCK300 (116×90 mm), Calibrite ColorChecker Passport DUO (125×90 mm), X-Rite ColorChecker Passport (130×90 mm), Charttu 24 ColorChecker S (108×64 mm), Danes-Picta BST 13 (203×60 mm – set of two long charts with grey and color patches, respectively), Kodak/Tiffen Q-13 (203×60 mm – set of two long charts with grey and color patches, respectively).

*Micro format charts:* ISA ColorGauge Micro Target (42×35 mm), ISA ColorGauge RezChecker Micro (48×41 mm, includes additional CLP gauge), Charttu 24 ColorChecker XS (50×70 mm), Charttu 24 ColorChecker XXS (45×40 mm).

*Nano format charts:* X-Rite ColorChecker Classic Nano (40×24 mm), Calibrite ColorChecker Classic Nano (38×25 mm), ISA ColorGauge Nano Target (18×21 mm).

*Pico format charts:* ISA ColorGauge Pico Target (11×10 mm).

*Color reference charts with CLP gauge (resolution test pattern):* ISA ColorGauge RezChecker Micro (48×41 mm), ISA Golden Thread Object-Level Target x0.5 (118×16 mm), Danes-Picta BST1 (142×60 mm).

**Target undercover:** Cutting mat Axent Pro 7905-A А2.

**PC:** Lenovo ThinkCentre M83 Intel i5 4570 3.20Ghz 16GB RAM 500GB HDD.

**Monitor:** Color-calibrated Asus ProArt PA248QV *or* Asus ProArt PA248CRV.

**Additional equipment and materials**

- Inverter power generator Fogo F4001iSE – to supply electrical power to equipment in a case of blackouts.
- Popwerbank X-Vinga 20000 *or* similar – for quick charging the portable devices.
- USB type C cord – to charge the portable devices.
- Multi-function printers Brother MFC-7320R *and* Brother DCP 7030 – to print labels, cards and other materials.
- Network filter APC PMF83VT-GR – to protect the devices from electrical overloads.
- Card reader Transcend TS-RDF5K – to read the SD cards.
- External driver ASUS TurboDrive BW-12D1S-U – to write and read Blu-Ray and DVD disks.
- Optical disks Blu-Ray Verbatim BD-R DL Hard Coat MABL (50 GB) – for long-term data and images preservation.
- Network router TP-Link TL-MR6400 N300 4G LTE Wi-Fi – for the internet connection through the mobile services in a case of blackouts.
- Short-stroke membrane keyboard Cougar Vantar AX Black – short-stroke keys allow to speed up the data input.
- Additional programmable keyboard Logitech G13 – it allows additional programming of 22 special characters, which speeds up the data input without switching keyboard layouts/languages.
- Programmable mouse A4Tech X87 (Maze) – it allows programming special keys to Ctrl+C and Ctrl+V combinations, which speeds up the data input.
- Table lamp (2 pcs) – for workplace lighting.
- Herbarium logo stamp – to place on herbarium sheets of all specimens.
- Stamp ‘Digitised’ – to place on herbarium sheets of all digitised specimens.
- Automatic numerator Optima 7 – to print specimen IDs on the herbarium sheets.
- Saber cutter A4 KW-Trio 13925 – to cut the herbarium labels, notae criticae, etc.
- Stickers Xerox 003R93177 (65 pcs per А4 sheet) – to print barcodes.
- Paper А2 80 g/m^2^ – to produce herbarium sheet covers.
- Paper А3 250 g/m^2^ – to produce herbarium sheets.
- Archival chlorine-free paper Xerox A4 Premier ECF, 80 g/m^2^ (003R91720) – to print herbarium labels.
- Paper Color Copy A4, 100 g/m^2^ (411965) *or* Navigator Paper А4 Presentation 100 g/m^2^, class А (530232) – to print cards for the herbarium card-index.
- Glue PVP Axent *or* similar – to adhere herbarium labels and notae criticae.
- Self-adhesive water-activated paper tape (70 mm wide) – to mount the herbarium specimens on the herbarium sheets.

**Software**

- Adobe Photoshop CC 2018 + Camera Raw 12.2.1 – to process the images and convert the file formats.
- Adobe Photoshop Lightroom 5.7.1 – to process the images and convert the file formats.
- ColorChecker Camera Calibration 2.3.0 – to create calibration profile files DCP.
- Zint Barcode Studio 2.4.2 – to generate the barcodes and QR-codes.
- Adobe InDesign CC 2018 – to layout and print the labels and barcodes.
- Microsoft Excel 2016 – to create and fulfill the initial dataset and to convert the file formats.
- OpenRefine 3.7.6 – to clean the data and to apply the UTF-8 coding.
- ABBYY FineReader 11 – for the OCR processing of the herbarium labels.
- Barcode Editor 8.0.4239 – for bulk reading the barcodes from the images and renaming agreeably the respective images.
- BardecodeFiler (alternative) – for bulk reading the barcodes from the images and renaming agreeably the respective images.
- Microsoft Picture Manager 2010 (14.0.4750.1000) – to work with labels’ images during the data mobilisation (more recent versions are not convenient to work with).
- BurnAware 18.4 – to write the archival optical disks.
- LabelJoy 6 (optional) – for bulk layout and printing the herbarium labels.
- Sublime Text 3 (optional) – to work with textual data and basic codding.
- WmHelp XmlPad 3 (optional) – to work with XML files in table format.
- Foxit PDF Reader (optional) – to work with and annotate the PDF files.
- SySTools PDF Unlocker 3.0 (optional) – to unlock locked PDF files.
- Advanced Renamer 3.91 (optional) – for bulk file renaming.
- XnView MP (optional) – for basic processing of the images.
- XnConvert (optional) – alternative to Adobe Photoshop allowing extended image processing.

**Online sources**

- https://dwc.tdwg.org/ – Darwin Core, a reference guide.
- https://imagezebra.com/ – Image Zebra, an image quality (including ΔE_2000_) testing service.
- https://ipt.gbif.org/ – GBIF IPT (Integrated Publishing Toolkit), a service to publish the datasets on GBIF.
- <https://www.gbif.org/ipt> – GBIF IPT description and manual.
- https://www.gbif.org/tools/species-lookup – GBIF Backbone Taxonomy, a service to validate the taxonomy on conformance with that applied in GBIF.
- https://www.gbif.org/tools/data-validator – GBIF Data Validator, a service to validate the data in conformance with the GBIF requirements.
- http://resolver.globalnames.org/ – Global Names Resolver, a bulk taxonomic resolver.
- https://openherbarium.org/ – Open Herbarium, a service to store free the data of virtual herbarium.
- https://zenodo.org/ – Zenodo, an open data archiving and publishing service.
- https://www.openstreetmap.org/ – OpenStreetMap, an open service for working with geographic coordinates and maps.
- https://powo.science.kew.org/ – Plants of the World Online (POWO), a principal taxonomic check-list.
- https://www.worldplants.de/ – World Plants, an extremely detailed alternative taxonomic check-list.
- https://ipni.org/ –International Plant Names Index (IPNI), a principal service to check the nomenclature and search for standartised names of collectors.
- https://www.biodiversitylibrary.org/ – Biodiversity Heritage Library (BHL), a digital library, useful to search for the protologues.
- https://www.zobodat.at/ – Zobodat, a service useful to search for the protologues and data regarding botanists (first of all Austrian).
- https://www.wikidata.org/ – Wikidata, a service useful to search for the data about botanists.
- https://kiki.huh.harvard.edu/databases/botanist_index.html – Harvard Index of Botanists, a service useful to search for the data about botanists.
- https://bionomia.net/ – Bionomia, a unique service representing the data about the natural history collections and collectors.
- https://extendsclass.com/uuid-generator.html – a bulk UUID generator.
- https://www.google.com/maps (optional) – Google Maps, a service that can be useful as an additional source for georeferencing.
- https://transkribus.ai/ (optional) – Transkribus, an AI-based service for recognising handwritings.
- https://lens.google/ (optional) – Google Lense, an AI-based service for recognising images (including text on images).
- https://www.catalogueoflife.org/ (optional) –Catalogue of Life (CoL), a principal service to verify the higher taxonomy.
- https://www.jacq.org/ (optional) – JACQ, a consortium of virtual herbaria.
- https://ukrbin.com/ (optional) – Ukrainian Biodiversity Information Network (UkrBin), a database on biodiversity of Ukraine and other countries.
- http://gnrd.globalnames.org/ (optional) – Global Names Finder, a taxonomic names parser.
- https://www.gbif.org/tools/name-parser (optional) – GBIF Name Parser, a taxonomic names parser.
- https://scientific-collections.gbif.org/ (optional) – Global Registry of Scientific Collections (GRSciColl), a service to search the collections and validate their data (e.g., acronyms).
- https://sweetgum.nybg.org/science/ih/ – Index Herbariorum, an index of the world herbaria to search and validate their data (e.g., acronyms).
